# Quantitative comparison of the spreading and invasion of radial growth phase and metastatic melanoma cells in a three-dimensional human skin equivalent model

**DOI:** 10.7717/peerj.3754

**Published:** 2017-09-05

**Authors:** Parvathi Haridas, Jacqui A. McGovern, Sean D.L. McElwain, Matthew J. Simpson

**Affiliations:** 1 Institute of Health and Biomedical Innovation, Queensland University of Technology, Brisbane, Queensland, Australia; 2 School of Mathematical Sciences, Queensland University of Technology, Brisbane, Queensland, Australia

**Keywords:** Melanoma, Cancer, Skin cancer, Three dimensional model, Invasion, Skin model, Skin equivalent model, Metastasis, Cell migration, Cell line

## Abstract

**Background:**

Standard two-dimensional (2D) cell migration assays do not provide information about vertical invasion processes, which are critical for melanoma progression. We provide information about three-dimensional (3D) melanoma cell migration, proliferation and invasion in a 3D melanoma skin equivalent (MSE) model. In particular, we pay careful attention to compare the structure of the tissues in the MSE with similarly-prepared 3D human skin equivalent (HSE) models. The HSE model is identically prepared to the MSE model except that melanoma cells are omitted. Using the MSE model, we examine melanoma migration, proliferation and invasion from two different human melanoma cell lines. One cell line, WM35, is associated with the early phase of the disease where spreading is thought to be confined to the epidermis. The other cell line, SK-MEL-28, is associated with the later phase of the disease where spreading into the dermis is expected.

**Methods:**

3D MSE and HSE models are constructed using human de-epidermised dermis (DED) prepared from skin tissue. Primary fibroblasts and primary keratinocytes are used in the MSE and HSE models to ensure the formation of a stratified epidermis, with a well-defined basement membrane. Radial spreading of cells across the surface of the HSE and MSE models is observed. Vertical invasion of melanoma cells downward through the skin is observed and measured using immunohistochemistry. All measurements of invasion are made at day 0, 9, 15 and 20, providing detailed time course data.

**Results:**

Both HSE and MSE models are similar to native skin *in vivo*, with a well-defined stratification of the epidermis that is separated from the dermis by a basement membrane. In the HSE and MSE we find fibroblast cells confined to the dermis, and differentiated keratinocytes in the epidermis. In the MSE, melanoma cells form colonies in the epidermis during the early part of the experiment. In the later stage of the experiment, the melanoma cells in the MSE invade deeper into the tissues. Interestingly, both the WM35 and SK-MEL-28 melanoma cells lead to a breakdown of the basement membrane and eventually enter the dermis. However, these two cell lines invade at different rates, with the SK-MEL-28 melanoma cells invading faster than the WM35 cells.

**Discussion:**

The MSE and HSE models are a reliable platform for studying melanoma invasion in a 3D tissue that is similar to native human skin. Interestingly, we find that the WM35 cell line, that is thought to be associated with radial spreading only, is able to invade into the dermis. The vertical invasion of melanoma cells into the dermal region appears to be associated with a localised disruption of the basement membrane. Presenting our results in terms of time course data, along with images and quantitative measurements of the depth of invasion extends previous 3D work that has often been reported without these details.

## Introduction

Melanoma is a deadly form of skin cancer (Bertolotto, 2013; Weinstein et al., 2014) that is caused by the malignant transformation of melanocytes in the skin (Uong & Zon, 2010; Bertolotto, 2013; Liu, Peng & Tobin, 2013). Melanoma accounts for less than 10% of all skin cancers, however it is associated with 80% of skin cancer related deaths (Bandarchi et al., 2010; Bertolotto, 2013; Ramaraj & Cox, 2014; Leight et al., 2015; McCusker et al., 2017; Rivas et al., 2017). The early stage of a primary melanoma, where cancer cells are generally confined to the epidermis, is known as the radial growth phase (RGP) (Clark, 1991; Meier et al., 2000). Melanoma in the RGP is curable through surgical removal (Weinstock, 2000; Cummins et al., 2006). However, survival rates of patients with melanoma at a more advanced stage, where cancer cells have invaded vertically into the dermis, known as the vertical growth phase (VGP), is between 53 and 97%. The five-year survival time for VGP melanoma depends on the stage of the disease. In comparison with VGP melanoma, survival rates of patients with metastatic melanoma, where cancer cells have moved into the blood stream and away from the primary location is between 15 and 75%, depending on the stage of melanoma (Miller & Mihm, 2006; Sandru et al., 2014). The switch in progression, from radial spreading to vertical invasion is poorly understood (Hussein, 2004; Baruthio et al., 2008; Grahovac, Becker & Wells, 2013). Some cell lines are thought to be associated with the RGP (Bani et al., 1996; Cummins et al., 2006), whereas other cell lines are associated with more advanced stages of the disease (Fofaria & Srivastava, 2014; Tiwary et al., 2014). Thus, quantitative measurements of spreading and invasion of both RGP and metastatic cell lines in a 3D human skin model could help improve our understanding of melanoma progression, and the characteristics of both radial and vertical spreading.

Previous studies about the spreading of melanoma have focused on examining the spatial extent of population expansion, cell migration, cell proliferation, cell-to-cell adhesion and protein-expression on two-dimensional (2D) surfaces (Alexaki et al., 2010; Simpson et al., 2013; Treloar et al., 2013; Treloar et al., 2014). These 2D studies are straightforward to perform and cost effective (Beaumont, Mohana-Kumaran & Haass, 2014; Johnston, Simpson & McElwain, 2014; Binny et al., 2016). Moreover, 2D models can be used for preliminary co-culture investigations to examine potential interactions between different cell types (Beaumont, Mohana-Kumaran & Haass, 2014; Haridas et al., 2017). This flexibility is very important for melanoma research as co-culture assays are more realistic than monoculture assays since co-cultures allow melanoma cells to interact dynamically with other relevant cells, such as fibroblasts and keratinocytes (Gaggioli & Sahai, 2007; Li, Fan & Houghton, 2007; Beaumont, Mohana-Kumaran & Haass, 2014; Sriram & Bigliardi-Qi, 2015).

Traditional 2D assays do not recreate a physiological environment similar to native human skin *in vivo* (Beaumont, Mohana-Kumaran & Haass, 2014). Perhaps the most obvious limitation of 2D experiments is that they cannot be used to quantify vertical invasion (Van-Kilsdonk et al., 2010; Vorsmann et al., 2013; Kramer et al., 2013; Taloni et al., 2014). To improve our understanding of the differences between radial and vertical invasion, it is of interest to make time course observations and measurements of the spreading and invasion of melanoma in a three-dimensional (3D) skin model (Brandner & Haass, 2013). Experimental studies focusing on melanoma spreading and invasion in 3D skin-based models have been described over the last 20 years. Table 1 compares key properties of some previous 3D skin-models using de-epidermised dermis (DED) to study melanoma progression and invasion. While other previous 3D models have been used, such as collagen-based models (Vorsmann et al., 2013), the brief review in Table 1 is restricted to those previous studies explicitly using 3D-DED models.

**Table 1:** Key features of previous 3D-DED melanoma skin model studies. Key properties of previous studies using 3D-DED to establish HSE and MSE models. *Kc* indicates primary keratinocyte cells, and *Fb* indicates primary fibroblast cells. *RGP*, *VGP* and *metastatic* indicates studies that have used cell lines associated with these melanoma phases.

Previous studies	Melanoma cell lines included			Comparison of MSE and HSE structure		Basement membrane marker	Proliferation marker	Migration marker	Melanoma marker	Measurements of invasion depth	Time course images
	RGP	VGP	Metastatic	Kc	Fb						
Bechetoille et al. (2000)	No	No	Yes	Yes	No	Yes	No	Yes	Yes	No	No
Eves et al. (2000)	No	No	Yes	Yes	Yes	Yes	No	No	Yes	No	No
Dekker et al. (2000)	Yes	Yes	Yes	Yes	No	Yes	No	No	Yes	No	No
Mac Neil et al. (2000)	No	No	Yes	Yes	Yes	Yes	No	No	Yes	No	No
Eves et al. (2003b)	No	No	Yes	Yes	Yes	Yes	No	No	Yes	Yes	No
Eves et al. (2003a)	No	No	Yes	Yes	Yes	Yes	No	No	Yes	Yes	No
Dennhofer et al. (2003)	No	No	Yes	No	No	No	No	No	Yes	No	No
Marck et al. (2005)	No	No	Yes	Yes	Yes	Yes	No	No	Yes	Yes	No
Van-Kilsdonk et al. (2008)	No	No	Yes	Yes	No	Yes	No	No	Yes	No	No
Yang, Sule-Suso & Sockalingum (2008)	No	No	Yes	Yes	Yes	Yes	No	No	Yes	No	No
Van-Kilsdonk et al. (2010)	No	No	Yes	Yes	No	Yes	No	No	Yes	No	No
Marques & Mac Neil (2016)	No	No	Yes	Yes	Yes	Yes	No	No	Yes	Yes	No
Current study	Yes	Yes	Yes	Yes	Yes	Yes	Yes	Yes	Yes	Yes	Yes

**Figure 1:**
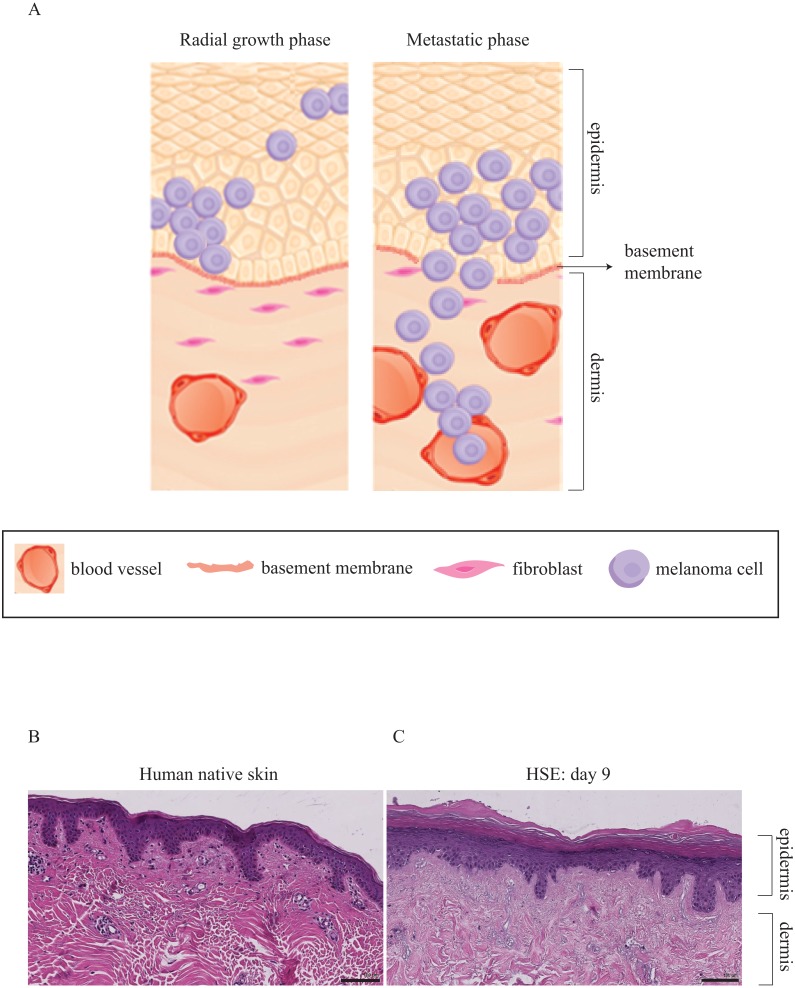
Three-dimensional representation of melanoma progression. (A) Schematic representation of the RGP phase, associated with melanoma cells in the epidermal region only and the metastatic phase, associated with melanoma cells that move away from the primary site. The cells in the metastatic phase are able to cross the basement membrane, enter the dermis and move into the blood vessels. This illustration is adapted, with permission, from Zaidi, Day & Merlino (2008). (B) and (C) H&E staining of native human skin and HSE respectively, showing a well-defined epidermis and dermis. The scale bar corresponds to 100 µm.

There has been extensive research focusing on 3D melanoma migration and spreading using cell lines that are associated with the metastatic phase of melanoma progression (Damsky, Rosenbaum & Bosenberg, 2010; Finn, Markovic & Joseph, 2012; Tiwary et al., 2014). However, in this work we are also interested in the differences between: (i) radial migration, where melanoma cells are confined to the epidermis and associated with the early phase of melanoma progression; and (ii) vertical invasion that is associated with more advanced melanoma progression. Therefore, we quantitatively compare the vertical invasive properties of two melanoma cell lines in a 3D skin model as a function of time. In particular, we compare results from one cell line that is associated with the early RGP stage of melanoma progression with results from another cell line that is linked with a more advanced, metastatic stage of the disease. A schematic illustrating the key differences between RGP and metastatic stages of the disease are given in Fig. 1A.

VGP melanoma is an intermediate phase of the disease that is thought to be less aggressive than the metastatic phase (Hsu et al., 1998; Meier et al., 2000; Satyamoorthy et al., 1997). However, as RGP melanoma is generally thought to be confined to the epidermis, we think that the VGP phase is more aggressive than RGP. Therefore, in this study we compare RGP and metastatic cell lines only since we aim to investigate the differences between these phases and it is reasonable to assume that these differences will be more obvious by comparing the invasion of cells that are thought to be associated with the most aggressive phase of the disease with cells that are thought to be associated with a less aggressive phase of the disease. In addition, we anticipate that a cell line associated with the VGP would produce results that are intermediate between the RGP and metastatic results.

Overall, we hypothesise that our MSE model recreates both the spatial and temporal distribution of melanoma cells as observed in native human skin *in vivo*. Our approach is novel because this study extends previous 3D melanoma studies summarised in Table 1, as we compare results from RGP and metastatic cell lines, providing quantitative measurements of melanoma cell invasion in a time course.

Previous studies demonstrate particular protocols of DED to construct human skin equivalent (HSE) models (Xie et al., 2010; Fernandez et al., 2014; McGovern et al., 2013). These 3D skin models are established *in vitro* and resemble native human skin *in vivo* as shown in Figs. 1B and 1C. One of our aims in this study is to adapt this skin model and introduce melanoma cells to establish a sustainable melanoma skin equivalent (MSE) model and recreate the different stages of melanoma progression. The other primary aim is to make quantitative measurements of the depth of melanoma invasion as a function of time, and to use these measurements to examine differences between the two cell lines that we consider.

Two melanoma cell lines, WM35 (RGP) (Herlyn, 1990) and SK-MEL-28 (metastatic phase) (Carey et al., 1976) are grown in the MSE model over a period of 9, 15 and 20 days. We identify differences in behavior between the two cell lines, and in particular we quantify the vertical invasion of melanoma cells into the dermis over time. The conclusions facilitate an improved characterisation of MSE models, and the progression of RGP and metastatic phases of melanoma in realistic 3D environments, thereby extending previous 2D studies.

## Experimental Methods

### Keratinocyte isolation and culture

Queensland University of Technology (QUT) human research ethics provides written approval for the skin samples to be used in this study (approval number: QUT HREC #1300000063; UnitingCare Health 2003/46). The samples come from patients undergoing abdominoplasty surgery and breast reduction surgery (Xie et al., 2010).

Human keratinocyte cells are isolated from skin and cultured in full Green’s medium following protocols described in Rheinwald & Green (1975), Dawson et al. (2006) and by Haridas et al. (2016). Primary keratinocyte cells are cultured at 37 °C, in 5% CO_2_ and 95% air.

### Fibroblast isolation and culture

Human fibroblast cells are isolated following protocols in Haridas et al. (2017). Primary fibroblast cells are cultured at 37 °C, in 5% CO_2_ and 95% air.

### Melanoma cell culture

The human melanoma cell lines, WM35 and SK-MEL-28 are cultured as described in Haridas et al. (2016). WM35 melanoma cells are kindly donated by Professor Nikolas Haass (University of Queensland Diamantina Institute) and SK-MEL-28 melanoma cells are donated by Professor Brian Gabrielli (Mater Research Institute-University of Queensland). Cells are cultured at 37 °C, in 5% CO_2_ and 95% air.

Both melanoma cell lines, WM35 and SK-MEL-28, are validated using short tandem repeat profiling (Cell Bank, Australia. January 2015). This means that the cell lines that we use are identical to the reference samples held in Cell Bank.

### Establishing HSE and MSE

**Figure 2:**
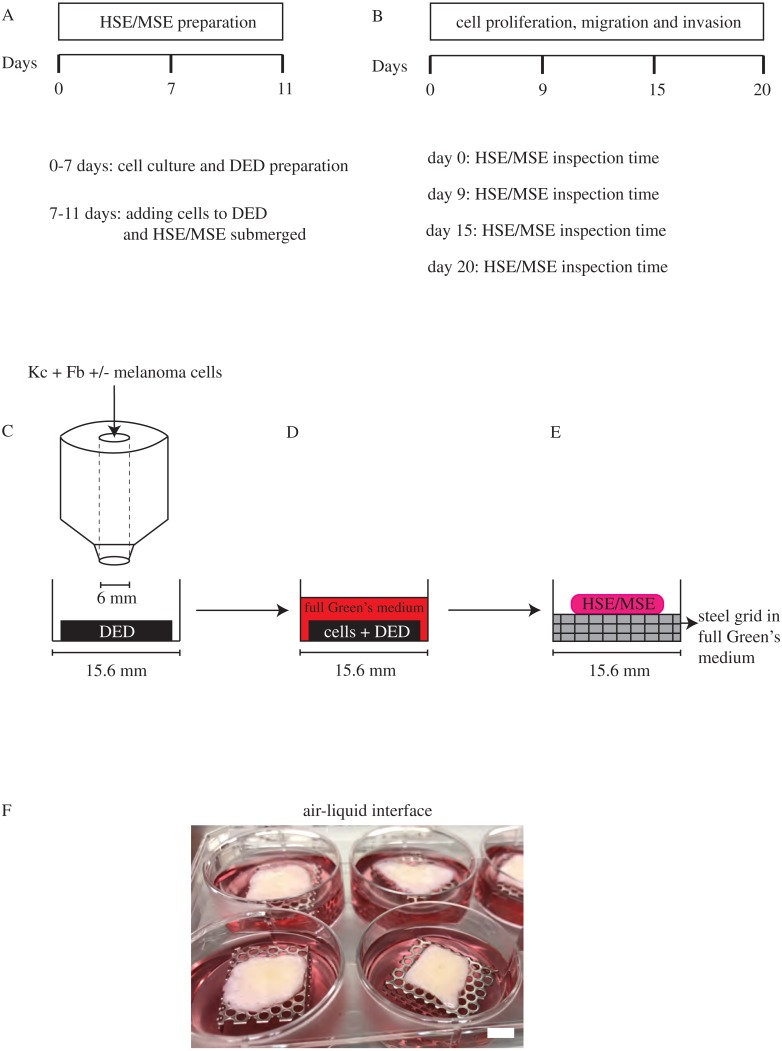
HSE and MSE preparation. (A) Time frame for cell culture and DED preparation to construct HSE and MSE models. (B) Time intervals at which the HSE and MSE models are cultured and inspected. (C) Schematic of the circular barrier assay showing how cells are placed inside the barrier on a DED within a 24-well tissue culture plate. (D) DED with cells submerged in full Green’s medium. (E)–(F) Schematic and image of the HSE and/or MSE models lifted to the air-liquid interface on a sterile stainless steel grid with full Green’s medium placed in a 6-well plate. Scale in (F) bar corresponds to 6 mm.

HSE models are established using the skin collected from donors undergoing elective plastic surgery. The protocol for establishing the HSE model is given in Figs. 2A–2F. These protocols are adapted from previous work (Fernandez et al., 2014; McGovern et al., 2013). The DED is prepared following protocols described by Chakrabarty et al. (1999) and Dawson et al. (2006). In brief, to construct the HSE model, sterile stainless steel rings (Aix Scientifics, Germany) with a diameter of 6 mm are placed on the papillary side of the DEDs in a 24 well tissue culture plate (Nunc®, Australia). Primary keratinocyte cells (2 × 10^4^) and primary fibroblast cells (1 × 10^4^) are seeded onto the DEDs in full Green’s medium and incubated at 37 °C, in 5% CO_2_ and 95% air for 2 days. Subsequently, the DEDs with cells, from now onwards referred to as HSE, is submerged in full Green’s medium for 2 days. These HSEs are then cultured at an air-liquid interface on sterile stainless steel grids with full Green’s medium for 9, 15 and 20 days. HSE is also collected at day 0, just before the DED is lifted to the air-liquid interface, as a reference sample.

To construct the MSE models, we follow the same protocol for the HSE model, and include melanoma cells, WM35 (5 × 10^3^) or SK-MEL-28 (5 × 10^3^), in addition to primary keratinocyte (2 × 10^4^) and primary fibroblast (1 × 10^4^) cells on the individual DEDs. This protocol of adding all the cells together on DEDs is standard in all the previous DED studies summarised in Table 1. Experimental variability is assessed using triplicates for each cell line, and primary skin cells from three separate donors. This means that for each time point in our experiments we perform nine replicates, which accounts for biological and experimental variability. HSEs and MSEs are collected after day 0, 9, 15, and 20, and subjected to histological investigation.

### MTT assay

An MTT (3-(4,5-dimethylthiazol-2-yl)-2,5-diphenyltetrazolium bromide) (Thermo Scientific) assay is performed to check the viability of cells in the HSE and MSE models. HSE and MSEs collected on day 0, 9, 15 and 20 are submerged in 0.5 mg/ml w/v MTT solution and incubated at 37 °C, in 5% CO_2_ and 95% air for 90 min. The metabolically active cells cleave the tetrazolium salt into an insoluble purple formazan dye. The purple colour indicates metabolically active cells on the HSE and MSE models and these are imaged using a stereo microscope (Nikon SMZ 800) fitted with a Nikon digital camera.

### Histological analysis

Haemotoxylin and eosin (H&E) staining is used to characterise the tissue structure in the HSE and MSE models. MTT stained HSE and MSEs are fixed using 10% neutral buffered formalin (United Biosciences, Australia), processed in an automated vacuum tissue processor (Thermo Scientific, USA) and embedded in paraffin wax. All samples are sectioned to 5 µm thickness using a microtome (Leica RM2245; Leica Microsystems, Australia). All HSE and MSE samples are first visually examined to see the spatial extent of the MTT positive region. Then, each sample is divided using a sterile blade, through the centre of the MTT positive region. The two smaller samples of tissue are each embedded in paraffin wax. These smaller samples are then further sectioned into 5 µm thick tissue sections using a microtome. This procedure allows us to explore the depth of vertical invasion that is close to the centre of where the population of cells is initially placed in a circular barrier onto the DED. Furthermore, by examining the depth of vertical invasion in the various 5 µm thick sections, we can examine whether the depth of vertical invasion depends on the lateral position. In summary, we find that the patterns of vertical invasion appear to be independent of the lateral position. Overall, in each experiment, we examine approximately 80–120 sections that are 5 µm in thickness. This means that we examine the vertical invasion of melanoma cells within a region extending from the centre of the initial population to approximately 400–600 µm away from that centre.

Sections are first deparaffinised in 100% xylene and rehydrated in graded ethanol series of 100%, 90% and 70%, and followed by distilled water. These sections are incubated in Harris haematoxylin (HD Scientific, Australia) followed by differentiation with 1% acid alcohol, bluing with Scott’s tap water solution and counterstaining with alcoholic eosin (HD Scientific). H&E stained sections are dehydrated in 90% and 100% ethanol, cleared with 100% xylene and mounted on coverslips using Pertex® mounting medium (Medite, Germany). All stained sections are imaged using an Olympus BX41 microscope fitted with an Olympus digital camera (Micropublisher, 3.3RTV, QImaging; Olympus, Q-Imaging, Tokyo, Japan).

### Immunohistochemistry

Immunohistochemistry is performed on the paraffin-embedded (5 µm) sections. Paraffin embedded sections are deparaffinised and rehydrated as previously described in McGovern et al. (2013). HSE and MSE skin sections are subjected to heat-mediated antigen retrieval treatment using either sodium citrate buffer (pH 6.0) or EDTA buffer (pH 8.0) in a decloaking chamber (Biocare Medical, USA) as described in Table 2. All skin sections are washed in phosphate buffered saline followed by immunostaining using MACH 4™ Universal HRP polymer kit (Biocare Medical). The temperature and time varies for each marker, as outlined in Table 2. The primary antibody for each protein is diluted in DaVinci Green diluent (Biocare Medical) to concentrations specified in Table 2, and these sections are incubated with the primary antibody for the time specified in Table 2. All the sections are finally counterstained using Gill’s haematoxylin (HD Scientific), dehydrated, mounted and imaged as described in ‘Histological Analysis’.

**Table 2:** Primary antibody protocols. Details of the primary antibodies and the antigen retrieval method used to detect the basement membrane (Col IV); terminal epidermal differentiation (Loricrin); migration (Vimentin); proliferation (Ki-67); and invasion (S100).

Antibody	Primary antibody			Antigen retrieval method		
	Antibody type	Source	Dilution	Time and temperature	Buffer	Time and temperature
Collagen IV (Col IV)	Mouse	DKSH, Australia	1:50	1 h, 37°C	Sodium Citrate (pH6.0)	20 min, 80°C
Ki-67	Mouse	Sigma Aldrich, Australia	1:100	1 h, 37°C	EDTA (pH8.0)	30 min, 90°C
Loricrin	Rabbit	Dako, Australia	1:100	1 h, 37°C	EDTA (pH8.0)	5 min, 97°C
S100	Rabbit	Dako, Australia	1:3,000	1 h, 37°C	Sodium Citrate (pH6.0)	5 min, 95°C
Vimentin	Rabbit	Thermo Scientific, Australia	1:800	12 –24 h,4°C	Sodium Citrate (pH8.0)	20 min, 80°C

### Image analysis

We use ImageJ (Treloar & Simpson, 2013; Johnston, Simpson & McElwain, 2014; ImageJ, 2017) to measure the depth of melanoma cell invasion into the dermal region on the MSE models at different time points. The depth of melanoma invasion is taken to be the distance from the top of the dermis to the deepest region invaded by the melanoma cells, as shown in Figs. S1A–S1C.

## Results and Discussion

### MTT assay of HSE and MSE

**Figure 3:**
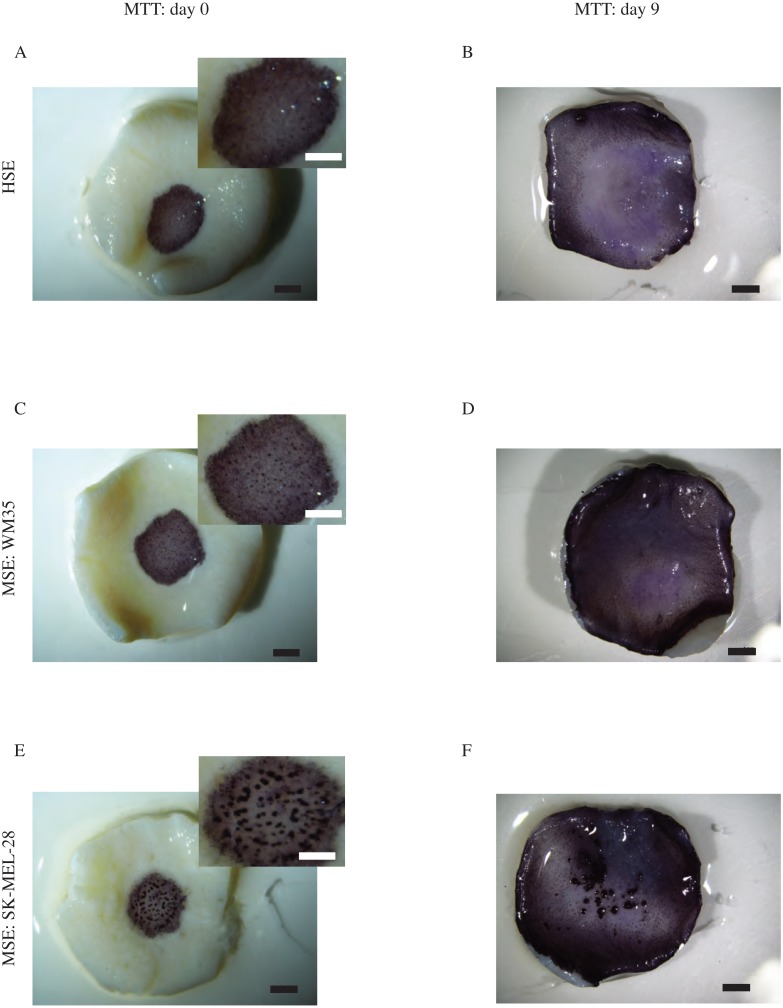
MTT assay. Experimental images of the MTT assay shows viable cells (purple) on the HSE (A)–(B). The MSE with WM35 melanoma cells is shown in (C)–(D). The MSE with SK-MEL-28 melanoma cells is shown in (E)–(F). Results in the left column are at day 0, and results in the right column are at day 9. The magnified central region of the HSE and MSE with melanoma cell colonies is shown in the insets in (A), (C) and (E). Scale bars in the main image show approximately 2 mm, whereas the scale bar in the insets show approximately 1.5 mm.

We first outline the MTT assay performed on both the HSE and MSE models. Results of the MTT assay, shown in Fig. 3, reveal radial expansion of the populations of cells on the HSE and MSE models over time. The purple colour on these images shows viable cells migrating radially away from the central region where the cells were originally located at day 0, as in Figs. 3A, 3C and 3E. By day 9, the cells have migrated radially to reach to edge of the DED, as in Figs. 3B, 3D and 3F. This means that the population of cells in the HSE and MSE have spread radially, at least a distance of approximately 6 to 7 mm, over a period of 9 days as the purple colouration reaches the edge of the tissue. Consistent with this, we see that there are viable cells distributed right across the DED in both the HSE and MSE models after longer periods of time, shown in Fig. S2.

An interesting result detected by the MTT assay is the formation of visually prominent colonies of cells in the central region of the MSE for the SK-MEL-28 cell line at day 0, as shown in the inset of Fig. 3E and also at day 9, as shown in Fig. 3F. Similarly, we also observe visually prominent colonies of cells in the central region of the MSE for the WM35 cell line at day 0, as shown in Fig. 3C. Previous 3D skin models of melanoma progression also report the formation of visually-distinct colonies of cells on the surface of the DED, and these colonies are presumably composed of melanoma cells (Dekker et al., 2000; Eves et al., 2000). Interestingly, we see that these distinct colonies of cells are no longer observed at day 15 or day 20 on the MSE with the SK-MEL-28 cell line, Fig. S2. Similarly, these distinct colonies of cells are no longer observed by day 9, 15 or 20 on the MSE with the WM35 cell line, Fig. 3D and Fig. S2. Since melanoma cells are thought to grow in colonies (Schwartz et al., 2008; Baraldi et al., 2013), a possible explanation for our observations is that the visually distinct dark purple colonies in the early period of the experiment could be groups of melanoma cells. As these colonies are not observed at later times, it is possible that these cells might have invaded deeper into the tissue, and are no longer present on the upper surface of the MSE. To confirm this conjecture, we now examine the distribution of different cell types within the HSE and MSE models. To do this we use histological analysis.

### The HSE and MSE physiology resembles native human skin *in vivo*

The next aim in our study is to examine the tissue structure of the HSE and MSE models, and to compare the structure of the tissue in these models with the structure of native human skin *in vivo*. To investigate this, we perform histological analysis and describe our results in this section, ‘Proliferation, Migration and Invasion of Melanoma Cells on the MSE Model’. However, we also use immunohistochemistry to examine the spatial and temporal distribution of markers for cell migration, cell proliferation and cell invasion in ‘Quantification of Melanoma Invasion’. Since the main focus of this work is about cell migration, cell proliferation and cell invasion, we choose to present all histological analysis about tissue structure in the Supplemental Information. However, we briefly describe the key points here.

Cross-sections through the HSE and MSE models are generated for H&E staining. Results at day 9, 15 and 20, showing HSE and MSE cross-sections, reveal morphological similarities to native human skin *in vivo.* In particular, we see the formation of distinct epidermal and dermal regions, Figs. S3B–S3D,  S4B–S4D and S5B–S5D. These images show that keratinocytes stratify into well-defined layers: stratum basale; stratum granulosum; stratum spinosum; and stratum corneum, which are a characteristic of native human skin (Wikramanayake, Stojadinovic & Tomic-Canic, 2014) as shown in Figs. 1B and 1C, Figs. S3A–S3D, S4A–S4D and S5A–S5D. However, H&E staining in Figs. S3A, S4A and S5A, of HSE and MSE cross-sections at day 0, are consistent with the early stages of epidermal and dermal formation, which then matures with time. In summary, we observe mature stratification after 9 days, and this is consistent with previous investigations (Topping et al., 2006).

The basement membrane separates the epidermal and dermal compartments, and is a prominent feature of native human skin *in vivo* (Marinkovich et al., 1993; Golan et al., 2015). The basement membrane is particularly important in the context of melanoma progression because melanoma confined to the epidermal compartment can be successfully treated by surgical removal, whereas the prognosis for melanoma that has spread into the dermis is poor (Weinstock, 2000; Cummins et al., 2006; Bertolotto, 2013; Sandru et al., 2014). The positive immunohistological staining is obtained using the marker collagen IV (Col IV). Immunohistological examinations of the HSE and MSE cross-sections show positive staining of the basement membrane at day 9, 15 and 20, as shown in Figs. S3F–S3H, S4F–S4H and S5F–S5H. However, all cross-sections of the skin models at day 0 show minimal positive staining. This is consistent with the initial development of the basement membrane, as highlighted by the arrows in Figs. S3E, S4E and S5E.

We observe a weakly stained, mostly-continuous basement membrane in skin models constructed using WM35 cell lines at day 9, as shown in Fig. S4F. Conversely, only intermittent Col IV staining is present in the MSE models with WM35 cells at day 15 and 20, as shown in Figs. S4G–S4H. Similarly, we observe intermittent Col IV staining in the MSE models with SK-MEL-28 cells at day 9, 15 and 20, as shown in Figs. S5F–S5H. Although the Col IV staining is relatively weak in these images compared to other staining results, we hypothesise that the Col IV staining results could be caused by melanoma cells disrupting the basement membrane and invading into the dermal region. Metastatic melanoma cells in particular are associated with dermal invasion *in vivo* by disturbing the basement membrane (Golan et al., 2015; Sandri et al., 2016). Therefore, this result further suggests that the MSE models recapitulates certain *in vivo* stages of melanoma progression *in vitro*.

Lastly, positive staining of the terminally differentiating epidermis confirms that both the HSE and MSE models constructed *in vitro* are similar to native human skin *in vivo*. The marker loricrin identifies terminally differentiating cells in the epidermis (Nithya, Radhika & Jeddy, 2015). Therefore, loricrin staining of HSE and MSE cross-sections, as shown in Figs. S3J–S3L, S4J–S4L and S5J–S5L, at day 9, 15 and 20, suggest that the epidermal structure in the HSE and MSE models is consistent with native human skin. However, results at day 0 from cross-sections of HSE and MSE models, shown in Figs. S3I, S4I and S5I, do not have any positive loricrin staining. Loricrin is known to be absent on non-stratified epithelium (Nithya, Radhika & Jeddy, 2015). Hence, the negative result at day 0 is probably due to the absence of the stratum corneum on day 0, which is consistent with an immature epidermis.

In summary, the loricrin staining suggests that the physiology of the HSE and MSE models is consistent with native skin. Furthermore, our findings show that HSE models have well-defined stratified epidermal and dermal regions that are separated by a basement membrane. This confirms that the *in vitro* HSE model is consistent with native human skin *in vivo*. In contrast, the MSE models do not always have a well-defined basement membrane. At early times in the experiments we see that the basement membrane is formed and present in the MSE model. However, at later times, the basement membrane in the MSE model is partially absent. These differences between the MSE and HSE models suggest that the presence of melanoma cells in the MSE models might lead to disruptions in the basement membrane. Furthermore, we hypothesise that this disruption is associated with vertical invasion.

### Proliferation, migration and invasion of melanoma cells on the MSE model

Certain key features of cancer progression, including melanoma, are thought to be the proliferation, migration and invasion of cancer cells (Hanahan & Weinberg, 2000). Therefore, we aim to explore the spatial and temporal distributions of these features in the HSE and MSE models. In particular, we use specific markers for cell migration, cell proliferation and cell invasion in our 3D models.

The MTT assay provides information about the radial spreading of cells across the MSE model. In addition to radial spreading, we also aim to observe and quantify the vertical invasion of melanoma cells, and in particular we wish to focus on cell lines that are associated with both the early and later stages of melanoma progression. RGP melanoma is generally associated with melanoma cells confined to the epidermal region of the skin (Clark, 1991; Meier et al., 2000). Previous experimental studies demonstrate that cells from the RGP are restricted above the intact basement membrane (Dekker et al., 2000; Meier et al., 2000). Hence we use WM35 melanoma cell lines that are derived from the RGP as this cell line represents the early phase of melanoma. VGP melanoma is associated with cells that enter and proliferate in the dermal region of the skin (Clark, 1991; Hsu et al., 1998; Zaidi, Day & Merlino, 2008). Cells from the VGP are thought to cross the basement membrane from the epidermis into the dermis (Hsu et al., 1998; Beaumont, Mohana-Kumaran & Haass, 2014). Additionally, metastatic melanoma cells not only invade into the dermis, but also have the ability to enter the blood stream and can therefore move far away from the primary site, to distant tissues (Clark, 1991; Zaidi, Day & Merlino, 2008). Cells derived from the metastatic phase are generally thought to be far more aggressive than cells from either the RGP or the VGP (Satyamoorthy et al., 1997). To examine these differences in our study we choose to focus on two cell lines: the WM35 cell line is associated with the RGP, which is thought to be the less aggressive phase of melanoma; the other cell line that we examine is the SK-MEL-28 cell line, which is from the metastatic phase of melanoma, and is thought to be the more aggressive.

**Figure 4:**
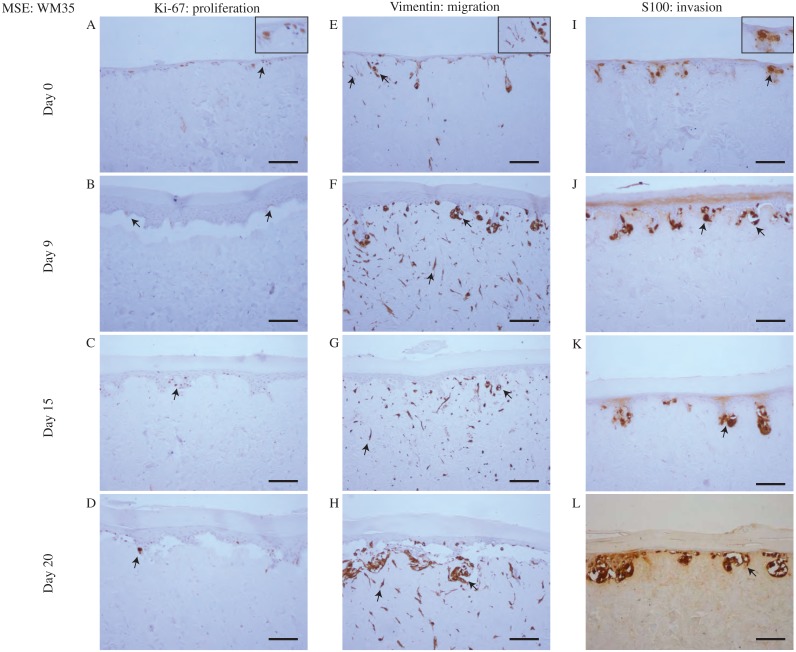
Proliferation, migration and invasion of skin cells and WM35 melanoma cells. (A)–(D) Proliferating cells (brown) highlighted by Ki-67 at day 0, 9, 15 and 20. (E)–(H) Migrating cells (brown) highlighted by vimentin. Dermal cells with elongated morphology are fibroblasts, and colonies of cells are migrating WM35 melanoma cells. (I)–(L) WM35 melanoma cells (brown) highlighted by S100 at day 0, 9, 15 and 20. Black arrows and inset images highlight positive staining. The scale bar in the main images shows 100 µm, and the width of the insets are approximately 75 µm.

**Figure 5:**
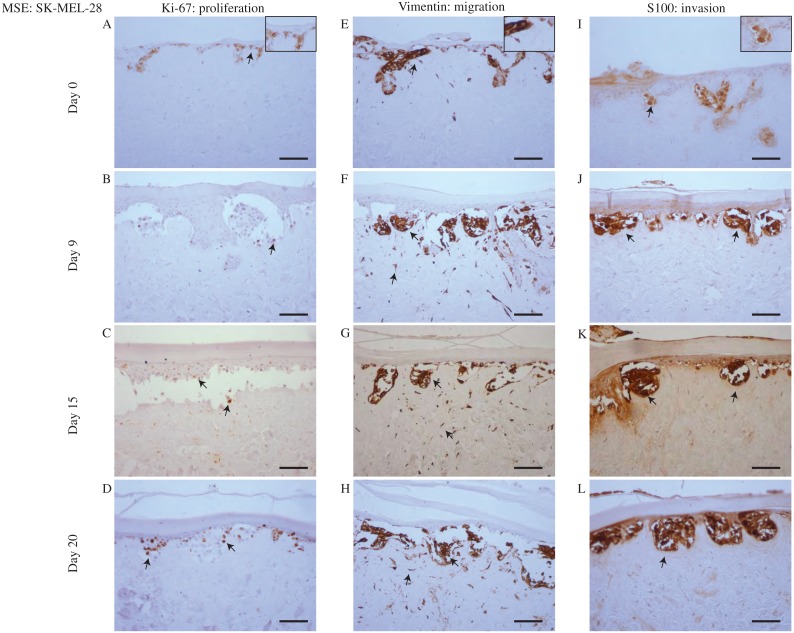
Proliferation, migration and invasion of skin cells and SK-MEL-28 melanoma cells. (A)–(D) Proliferating cells (brown) highlighted by Ki-67 at day 0, 9, 15 and 20. (E)–(H) Migrating cells (brown) highlighted by vimentin. Dermal cells with elongated morphology are fibroblasts, and colonies of cells are SK-MEL-28 melanoma cells. (I)–(L) SK-MEL-28 melanoma cells (brown) highlighted by S100 at day 0, 9, 15 and 20. Black arrows and inset images highlight positive staining. The scale bar in the main images shows 100 µm, and the width of the insets are approximately 75 µm.

To make this comparison we examine data from the MSE with the WM35 melanoma cell line in Fig. 4, with results using the SK-MEL-28 cell line in Fig. 5. Immunohistochemistry results in Figs. 4 and 5 indicate the migration, proliferation and invasion patterns of WM35 and SK-MEL-28 cell lines, respectively. We first identify actively proliferating cells in the MSE using the Ki-67 marker. Results in Figs. 4A–4D and 5A–5D highlight positively stained cells at day 0, 9, 15 and 20 for the WM35 and SK-MEL-28 cell lines, respectively. It is important to note that the Ki-67 marker identifies all proliferating cells, and does not distinguish between proliferating fibroblast cells, proliferating keratinocyte cells and proliferating melanoma cells. Therefore, additional information is required to distinguish between these different types of cells. Overall, we see that there are proliferative cells in both the epidermal and dermal regions of the tissue.

**Figure 6:**
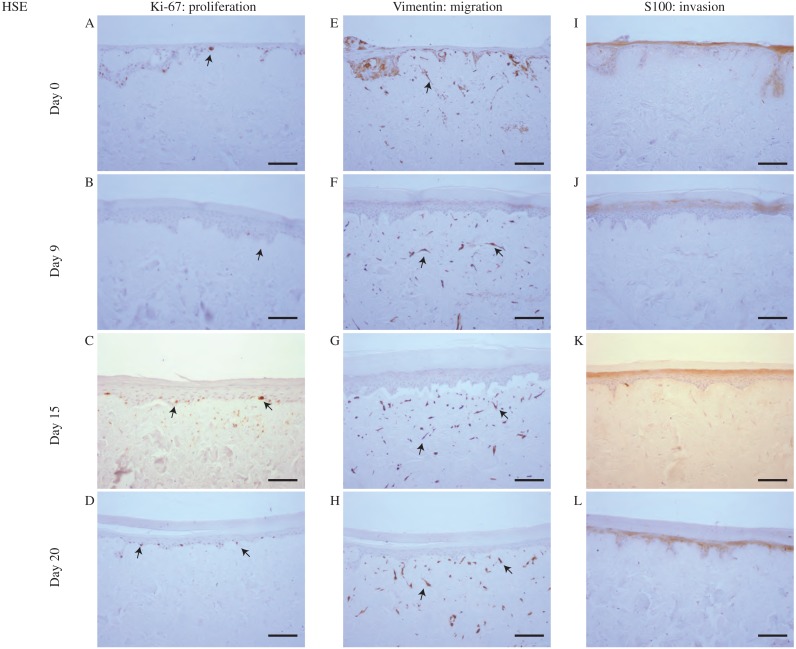
Proliferation, migration and invasion of skin cells. (A)–(D) Proliferating cells (brown) highlighted by Ki-67 at day 0, 9, 15 and 20. (E)–(H) Migrating fibroblast cells (brown) highlighted by vimentin. (I)–(L) No specific melanoma staining is highlighted by S100 at day 0, 9, 15 and 20. Black arrows and inset images highlight positive staining. Scale bar corresponds to 100 µm.

Migrating cells in the MSE models are detected using the marker vimentin (Ivaska et al., 2007; Chernoivanenko, Minin & Minin, 2013; Liu et al., 2015). In this context, migration is referred to motile fibroblast cells and motile melanoma cells. It is challenging to identify the particular type of migrating cells using vimentin, as vimentin is expressed by most mesenchymal cell types (Goodpaster et al., 2008; Chernoivanenko, Minin & Minin, 2013). Since both fibroblasts and melanoma cell lines are mesenchymal (Goodpaster et al., 2008; Kim et al., 2013; Sriram & Bigliardi-Qi, 2015) we expect that all melanoma and fibroblast cells will be positive for vimentin. To potentially distinguish between melanoma cells and fibroblast cells in the MSE models, we note that fibroblasts tend to be isolated and have an elongated cellular morphology (Sriram & Bigliardi-Qi, 2015). Furthermore, some of the vimentin positive cells appear to be arranged in colonies, and this is consistent with typical melanoma morphology (Schwartz et al., 2008; Kim et al., 2010; Baraldi et al., 2013). With this additional information, vimentin can be used to indicate the spatial distribution of fibroblasts, which appear to be only present in the dermal region, as shown in Figs. 4E–4H in the MSE with the WM35 melanoma cell line, and in Figs. 5E–5H in the MSE with the SK-MEL-28 melanoma cell line, at day 0, 9, 15 and 20. The fact that we tend to see fibroblast cells in the dermal region only provides further evidence that the MSE models resemble the HSE model, as shown in Fig. 6, as well as native human skin *in vivo* (Sriram & Bigliardi-Qi, 2015). Since fibroblast cells have migrated vertically downward, into the dermis, our MSE and HSE models also capture a key feature of native human skin, as fibroblasts are typically confined to the dermal region (Driskell & Watt, 2015). Note that in Fig. 6, all vimentin positive fibroblasts appear to be negative for S100. The fibroblasts are introduced into the DEDs along with keratinocyte cells and melanoma cells, which is 4 days before we collect our first results at the day 0 time point. We observe that there are more vimentin positive cells in the dermis on day 9 as shown in Fig. 6F, than on day 0, as shown in Fig. 6E. This indicates that the fibroblast cells have migrated vertically into the dermis. Vimentin positive melanoma cells, arranged in colonies, are detected in both the epidermal and dermal region of the MSE models, as shown in Figs. 4E–4H and 5E–5H, at day 0, 9, 15 and 20. All of the interpretations of the type of vimentin positive cells involve some subjective assessment of whether the cells are single, elongated or whether they appear to be arranged in colonies. To provide further information to distinguish between melanoma cells and fibroblast cells, we now use a specific marker for melanoma cells (Haridas et al., 2016).

Vertical invasion of melanoma cells into the MSEs is detected by the marker S100. Invasion in this context refers to vertical spreading of melanoma cells into the dermis. Our previous studies show that S100 is a reliable marker that identifies both the WM35 and the SK-MEL-28 melanoma cell lines (Haridas et al., 2016; Haridas et al., 2017). Since the vimentin marker detects all elongated and motile cells in this MSE model, the inclusion of the S100 marker specifically allows us to distinguish melanoma cells from fibroblast cells. In both MSE models with the WM35 cell line and the SK-MEL-28 cell line, colonies of melanoma cells are present at day 0, 9, 15 and 20. Individual cells within these colonies are positively stained by S100. Smaller colonies of melanoma cells are initially present near the upper surface of the MSE models, as shown in Figs. 4I and 5I. These melanoma colonies dramatically increase in size and number with time, and the melanoma colonies invade into the dermis after day 15 and 20, as shown in Figs. 4K and 4L for the WM35 melanoma cell line, and after day 9 in Figs. 5J–5L for the SK-MEL-28 melanoma cell line. It is important to note that on day 9, the WM35 melanoma cells are present only in the epidermal region. No S100 positive cells are present in the dermis. This observation, along with the Col IV staining of a mostly-continuous basement membrane at day 9, as shown in Fig. S4F, excludes the possibility of melanoma cells being trapped in the dermal region from the beginning of the experiment.

Comparing the size of the melanoma colonies over time in both MSE models shows that the colonies of SK-MEL-28 melanoma cells are larger than the colonies of WM35 melanoma cells. These differences are most evident at day 20, as shown by comparing the images in Figs. 4L and 5L. These results suggest that the SK-MEL-28 cell line is more aggressive than the WM35 melanoma cell line. This difference is consistent with the usual notion that the SK-MEL-28 melanoma cell line is associated with the later, more aggressive stage of the disease, whereas the WM35 melanoma cell line is associated with the early phase of melanoma progression. Also, it is possible that these immunohistochemistry results are consistent with the previous MTT results in Figs. 3E and 3F since the colonies of cells on the surface of the MSE model seem to disappear at later times. We initially observe colonies of cells, that we assume to be melanoma cells, on the surface of the MSE model on day 0 and day 9, as shown in Figs. 3E and 3F. It is reasonable to assume that these colonies are composed of SK-MEL-28 melanoma cells because there are no visible colonies on the equivalent HSE models at the same time points, as shown in Figs. 3A and 3B. These colonies are no longer visible on the MSE model after day 15, as shown in Fig. S2. Since we also observe S100 positive SK-MEL-28 cells moving vertically downward into the dermis over time, we believe that the MTT results of day 15 and day 20 are consistent with the S100 staining. That is, the eventual disappearance of the cell colonies on the surface of the MSE model could be a result of the melanoma cells moving deeper into the MSE tissue at later times.

To explore whether the differences in invasion of the two melanoma cell lines might be associated with any difference in cell size, we measure the size of WM35 and SK-MEL-28 cells, as shown in Fig. S6. These results show that the average size of both cell lines is approximately 10 µm. Therefore, the difference in invasion of the two cell lines is not attributed to any differences in cell size.

An interesting result from the MSE with the WM35 melanoma cell line is that we observe the invasion of small colonies of WM35 melanoma cells into the dermis at day 20, as shown in Fig. 4L. This result is interesting because WM35 melanoma cells are thought to be associated with the early phase of melanoma progression, where cells are believed to be limited to the epidermis (Gaggioli & Sahai, 2007). In Figs. 4H and 4L where WM35 melanoma cells are present in the dermis, we see intermittent staining of Col IV, suggesting that the basement membrane is somehow disrupted. In comparison, results in Fig. S4H where there are no melanoma cells present in the dermis, we see a more continuous Col IV staining, suggesting that the basement membrane is present and intact. These results, combined, are consistent with the notion that WM35 cells enter the dermis by somehow disrupting the basement membrane. While we have not investigated the mechanism by which the basement membrane is disrupted in detail, our conclusion that the WM35 cells appear to disrupt the basement membrane seems to be a plausible explanation of our results. Previous 3D studies suggest that cells from the early RGP are restricted in the epidermal region only (Dekker et al., 2000; Beaumont, Mohana-Kumaran & Haass, 2014). Therefore, our results are contradictory, suggesting that WM35 cells are able to breach the basement membrane and invade into the dermis in our MSE model.

Overall, these results showcase the successful establishment of a reliable and enduring MSE model that can be used to examine the migration, proliferation and invasion of melanoma cells from two different cell lines associated with RGP and metastatic stages of melanoma progression. However, in addition to providing qualitative information about the spatial and temporal distribution of different cell types in the MSE models, we also provide quantitative information about the invasion process.

### Quantification of melanoma invasion

**Figure 7:**
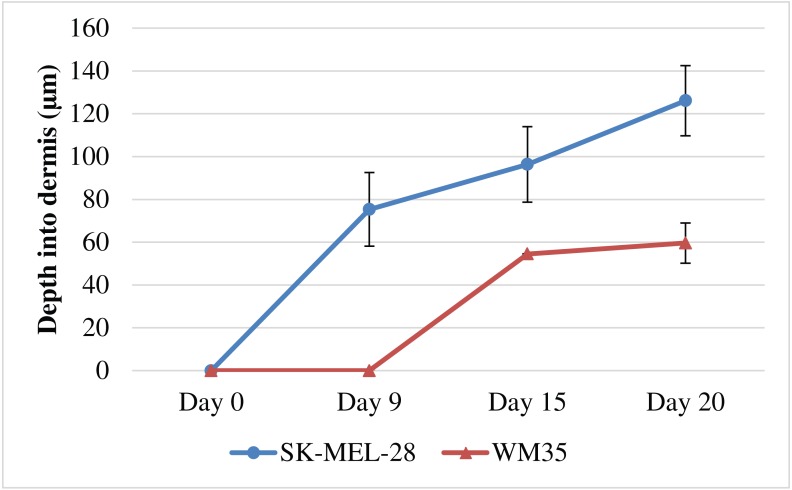
Quantification of melanoma cell invasion depth. Depth of melanoma invasion for the WM35 (red) and SK-MEL-28 (blue) cell lines. Data points show the average depth of invasion. The error bars measure the variability, as given by the sample standard deviation. In each case the sample mean and sample standard deviation is calculated using measurements from at least nine (*n* = 9) identically prepared experiments.

To further examine the differences in the invasion patterns associated with the WM35 and SK-MEL-28 melanoma cell lines, we measure the depth of cell invasion into the dermal region over time. The depth of invasion is taken to be the distance from the top of the dermis to the deepest region invaded by the melanoma cells, as shown in Figs. S1A–S1C. The invasion depth is measured in each experiment, at each time point, using ImageJ (ImageJ, 2017). Each measurement is repeated using three biological replicates for the DED, primary keratinocyte cells and primary fibroblast cells and the average depth is calculated by averaging the data across the three biological replicates performed in triplicates. Therefore, a total of nine individual data points are used to generate each averaged data point in Fig. 7.

Results in Fig. 7 show that the SK-MEL-28 melanoma cells invade earlier, deeper and faster than the WM35 melanoma cells. For example, at day 0 neither the SK-MEL-28 nor the WM35 melanoma cells appear to be in the dermis, even with minimal basement membrane present. However, by day 9, the SK-MEL-28 melanoma cells have invaded into the dermis, whereas the WM35 melanoma cells are still contained within the epidermis. The slope of the curve for WM35 cells in Fig. 7 is steeper between day 9 and day 15. We believe that the initial difference of melanoma invasion between the cell lines, WM35 and SK-MEL-28 is not related to cell viability. We present all our results in a time course pattern, this allows identification of melanoma cells using S100, from day 0 until day 20. Melanoma positive cells observed in Figs. 4I and 5I reveal similar results regardless of whether the MSE is initialised with WM35 cells or SK-MEL-28 cells. This suggests that melanoma cells are viable during the first few days after co-culture. Note that the standard deviation of the invasion depth for WM35 cells is very small since there is very little variation in our measurements.

Previous research has measured the invasion of melanoma cells into the dermis (Eves et al., 2003b; Eves et al., 2003a; Marques & Mac Neil, 2016). These studies use a semi-quantitative measurement of cell counts, showing various metastatic melanoma cells invading the dermal region. It is interesting to note that our study of melanoma invasion using MSE models differs from previous approaches, as shown in Table 1. We use a simple method of visual analysis and measurement of melanoma cell invasion into the dermal region. Most importantly, we provide time course measurements of melanoma cell invasion.

In summary our results suggest that the WM35 and the SK-MEL-28 melanoma cell lines both exhibit invasive properties and have the ability to enter the dermis in our model. This is interesting because the WM35 melanoma cell line is thought to be associated with the early phase of melanoma progression where the cells are confined to the epidermis. We do, however, observe differences in the invasive properties of the two cell lines. For example, the WM35 cells appear to take a long duration of time to enter the dermis than the SK-MEL-28 cell. Our study does not explain why the WM35 cells take a longer period of time to enter the dermis. However, we anticipate that these differences could have many explanations. For example, the disruption of the basement membrane could be driven by some kind of chemical signal, and the differences in the speed of invasion could be associated by differences in the production rates of such chemical signals. Exploring these ideas is a topic for future research. Overall, our qualitative observations and quantitative measurements suggest that the WM35 melanoma cell line is less invasive than the SK-MEL-28 melanoma cell line.

## Conclusion

In summary, 3D skin model studies are more realistic, and more closely resemble native human skin *in vivo* than 2D studies. HSE skin models constructed using DED are used in many research areas including wound healing and burn studies (Topping et al., 2006; Xie et al., 2010; Monsuur et al., 2016). Since the physiological architecture of the HSE model is similar to native human skin *in vivo* it can be adapted to study melanoma proliferation, migration and invasion patterns. Melanoma has various phases of progression and 2D models are limited since 2D models cannot be used to study vertical invasion. In contrast, 3D skin-based melanoma models can be used to study vertical invasion, as well as exploring how melanoma cells interact with surrounding cells and tissues.

In this study we develop an *in vitro* MSE model using cell lines from early and late phases of melanoma. The MSE model incorporates ether WM35 melanoma cells or SK-MEL-28 melanoma cells, as well as primary keratinocytes and primary fibroblasts. Our MSE models are capable of examining melanoma progression for up to 20 days, which is the longest time point we have analysed. Collectively, our results suggest that MSE models constructed *in vitro* have similar tissue structure to native human skin. The melanoma cells in the MSE models proliferate, migrate and invade into the dermis as observed in native human skin *in vivo*. However, these two cell lines from the RGP and metastatic phase of melanoma lead to different patterns of invasion. Importantly, the MSE models enable quantitative measurements of the invasive process to be made, and allow us to quantitatively compare the progression of the two different cell lines.

Lastly, it could be of interest to extend this work by using cell lines associated with the VGP state of the disease in the MSE and comparing these additional results to our current study. Additionally the MSE could also be further developed as a pre-clinical platform to investigate the effects of anti-melanoma drugs. Both melanoma cell lines used in the current study have *BRAF* mutations (Smalley et al., 2008; Boussemart et al., 2014; Fofaria et al., 2015), hence these cell lines could possibly be targeted using a number of drugs such as vemurafnib, dabrafenib, trametinib, or a combination of either these drugs (Jang & Atkins, 2013; Boussemart et al., 2014; Fofaria et al., 2015). The alteration in cell proliferation, cell invasion and colony formation, when melanoma cells are treated with these putative drugs could be examined in this MSE model.

##  Supplemental Information

10.7717/peerj.3754/supp-1 Supplemental Information 1 Additional results Additional results
