# Supplementary material for: Quantitative comparison of the spreading and invasion of radial growth phase and metastatic melanoma cells in a three-dimensional human skin equivalent model
Source: PeerJ. 2017 Sep 5;5:e3754. doi: 10.7717/peerj.3754 (PMC5590551; doi:10.7717/peerj.3754)
Supplement: Supplemental Information 1 — Additional results [file peerj-05-3754-s001.pdf]

Supplemental Information

Quantitative comparison of the spreading and invasion of radial growth phase and metastatic melanoma cells in a three-dimensional human skin equivalent model

Parvathi Haridas[1,2], Jacqui A McGovern[1], D L Sean McElwain[1,2], Matthew J Simpson[1,2]

[1] Institute of Health and Biomedical Innovation, Queensland University of Technology (QUT), Brisbane, Australia.

[2] School of Mathematical Sciences, QUT, Brisbane, Australia.

Corresponding Author:

Matthew J Simpson[1,2]

QUT, Brisbane, Australia

Email address: matthew.simpson@qut.edu.au

Quantifying depth of melanoma cell invasion

Images in Fig. S1 shows how melanoma invasion depth measurements are derived from MSE models at day 9, 15 and 20. SK-MEL-28 cells are detected using the marker S100 as shown in Fig. S1A-S1C. This allows visualisation of melanoma cells in the MSE model. The depth of melanoma cell invasion is taken as the distance from the top of the dermis to the deepest region invaded by the melanoma cells. We measure the depth of invasion at day 9, 15 and 20, using ImageJ software (https://imagej.nih.gov/ij/docs/guide/146-29.html). Each measurement is repeated using three biological replicates for the DED, primary keratinocyte cells and primary fibroblast cells and each experiment is performed in triplicates. Therefore, the average depth is calculated by averaging the data obtained from n=9 experimental results.

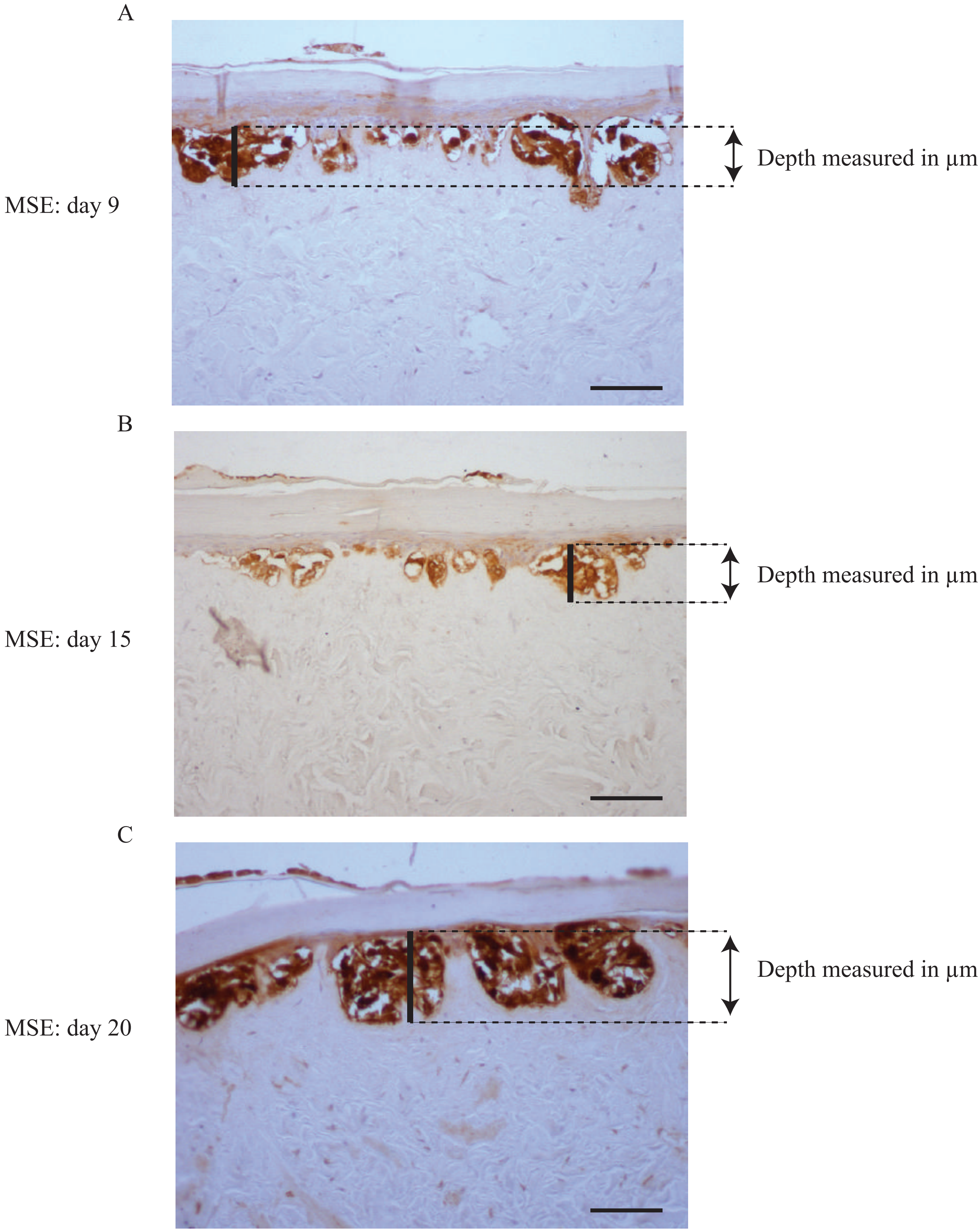
A
MSE: day 9
Depth measured in µm
B
MSE: day 15
Depth measured in µm
C
MSE: day 20
Depth measured in µm

Figure Caption S1: Quantifying depth of melanoma cell invasion.

(A)-(C) MSE model with SK-MEL-28 melanoma cells (brown) highlighted by S100 at day 9, 15 and 20. The black arrows indicate the depth of melanoma cell invasion measured. Scale bar corresponds to 100 µm.

MTT assay

Viable cells (purple) are identified in the HSE model, MSE model with WM35 cells and MSE model with SK-MEL-28 cells using MTT assay. Images in Fig. S2 show MTT assay results obtained at day 15 and 20. These images reveal the radial expansion of the total cell population on the HSE and MSE models imaged using a stereo microscope fitted with a Nikon digital camera.

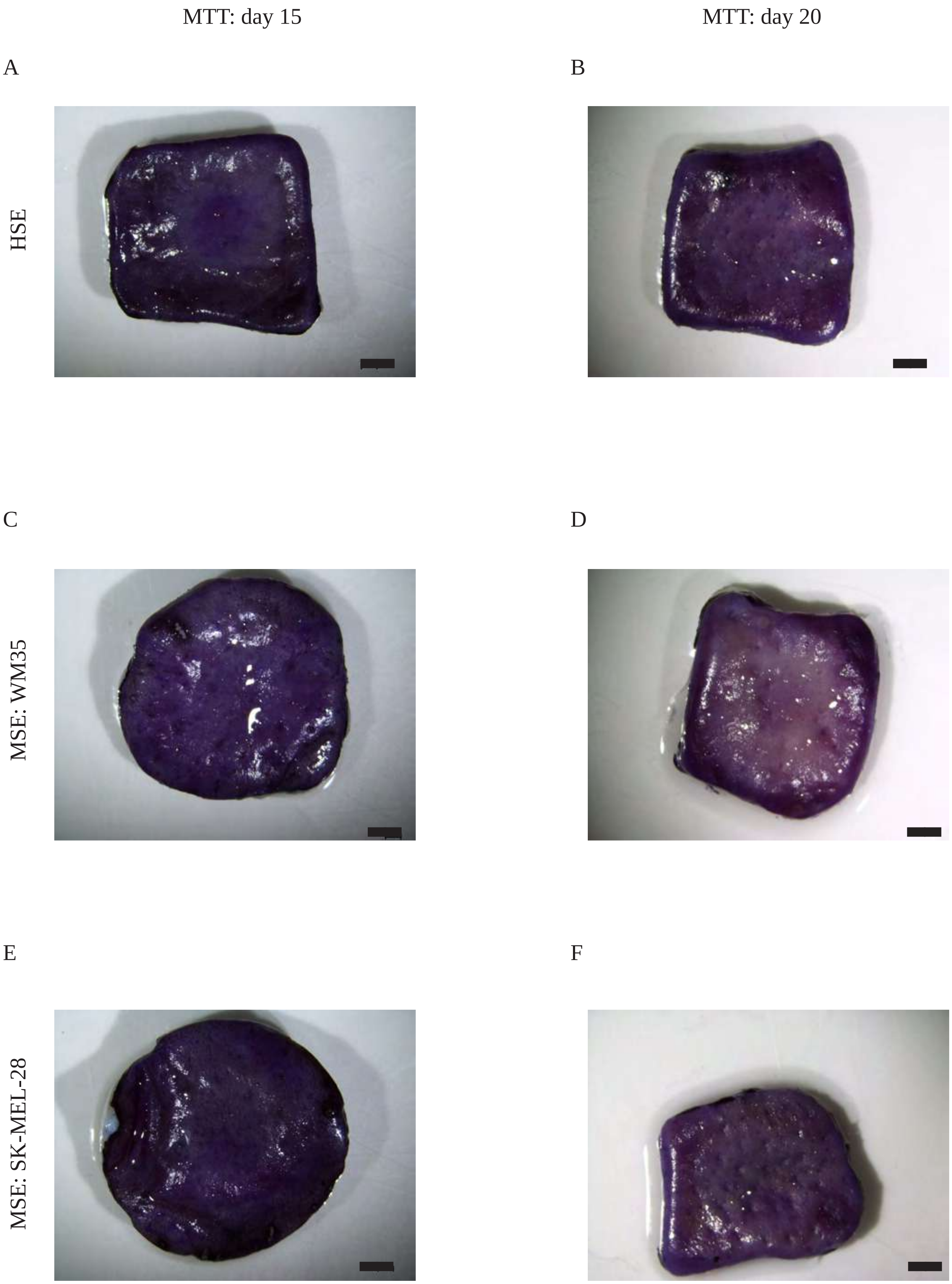
MTT: day 15
MTT: day 20
A
B
HSE
C
D
MSE: WM35
E
F
MSE: SK-MEL-28

Figure Caption S2: MTT assay.

Experimental images of MTT assay shows viable cells (purple) on the HSE model (A) and (B).The MSE model with WM35 melanoma cells is shown in (C) and (D).The MSE model with SK-MEL-28 melanoma cells is shown in (E) and (F). The results in the left column are at day 15, and the results in the right column are at day 20. Scale bar corresponds to 1 mm.

Histological analysis of HSE model

HSEs are first divided through the centre of the MTT positive region using a sterile blade and embedded in paraffin wax. The samples are then sectioned using a microtome into 5 µm thick tissue sections. To show the physiological similarities of the HSE model to native skin we perform H&E staining, basement membrane staining using Col IV, and terminally differentiating epidermal cell staining using Loricrin, on these tissue sections, as shown in Fig. S3. Staining is performed at day 0, 9, 15 and 20 and the results are imaged using Olympus BX41 microscope fitted with an Olympus digital camera.

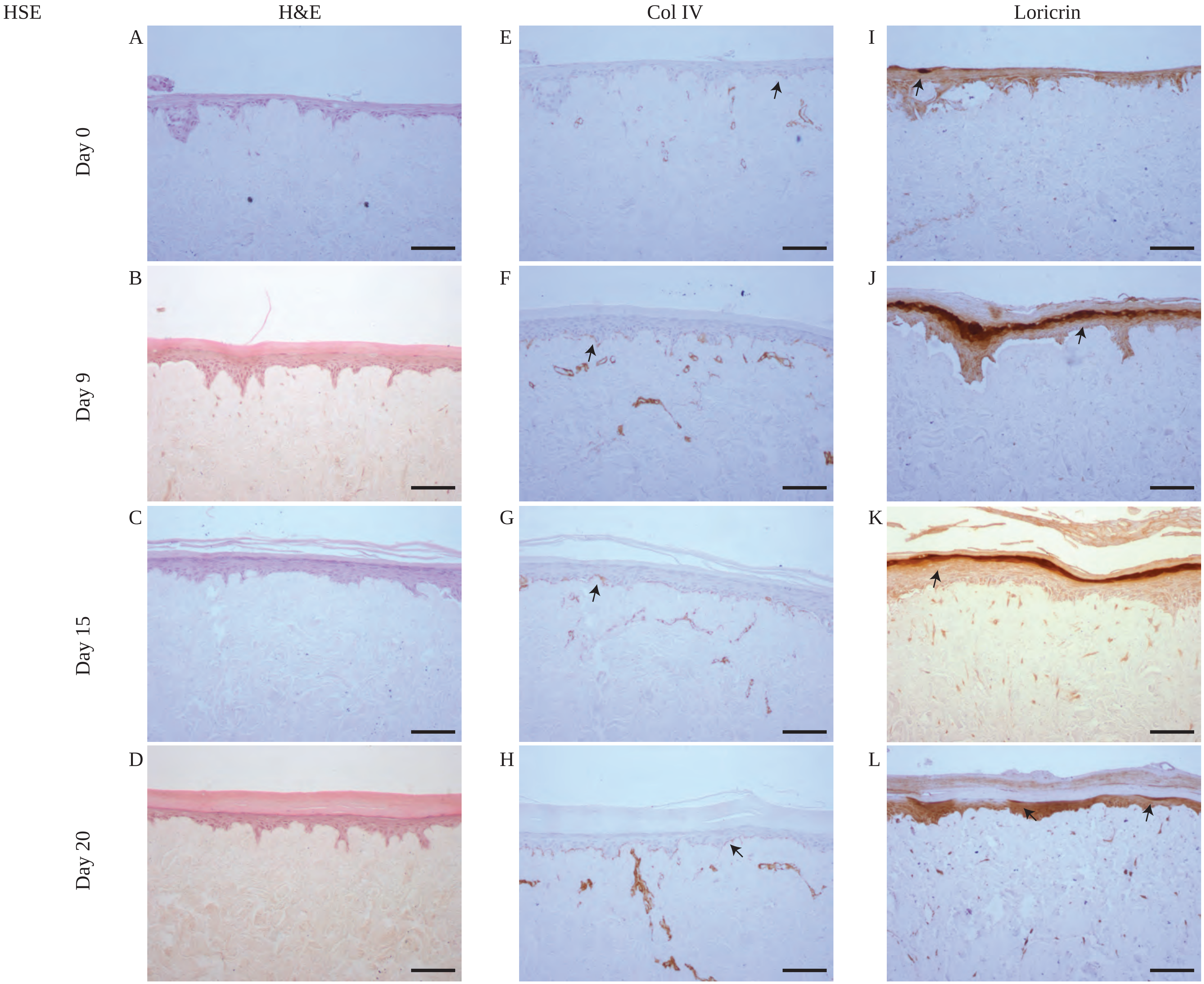
HSE
H&E
Col IV
Loricrin
Day 0
Day 9
Day 15
Day 20
A
B
C
D
E
F
G
H
I
J
K
L

Figure Caption S3: Histological analysis of HSE model.

(A)-(D) H&E staining at day 0, 9, 15 and 20. (E)-(H) The basement membrane (brown) highlighted by collagen IV (Col IV) at day 0, 9, 15 and 20. (I)-(L) Terminally differentiating epithelial cells (brown) highlighted by loricrin at day 0, 9, 15 and 20. Black arrows indicate positive staining. Scale bar corresponds to 100 µm.

Histological analysis of MSE model with WM35 melanoma cells

MSE models with WM35 cells are first divided through the centre of the MTT positive region using a sterile blade and embedded in paraffin wax. The samples are then sectioned into 5 µm thick tissue sections. To show the physiological similarities of the MSE model to HSE and native skin we perform H&E staining, basement membrane staining using Col IV, and terminally differentiating epidermal cell staining using Loricrin, on these tissue sections, as shown in Fig. S4. Staining is performed at day 0, 9, 15 and 20 and the results are imaged using Olympus BX41 microscope fitted with an Olympus digital camera.

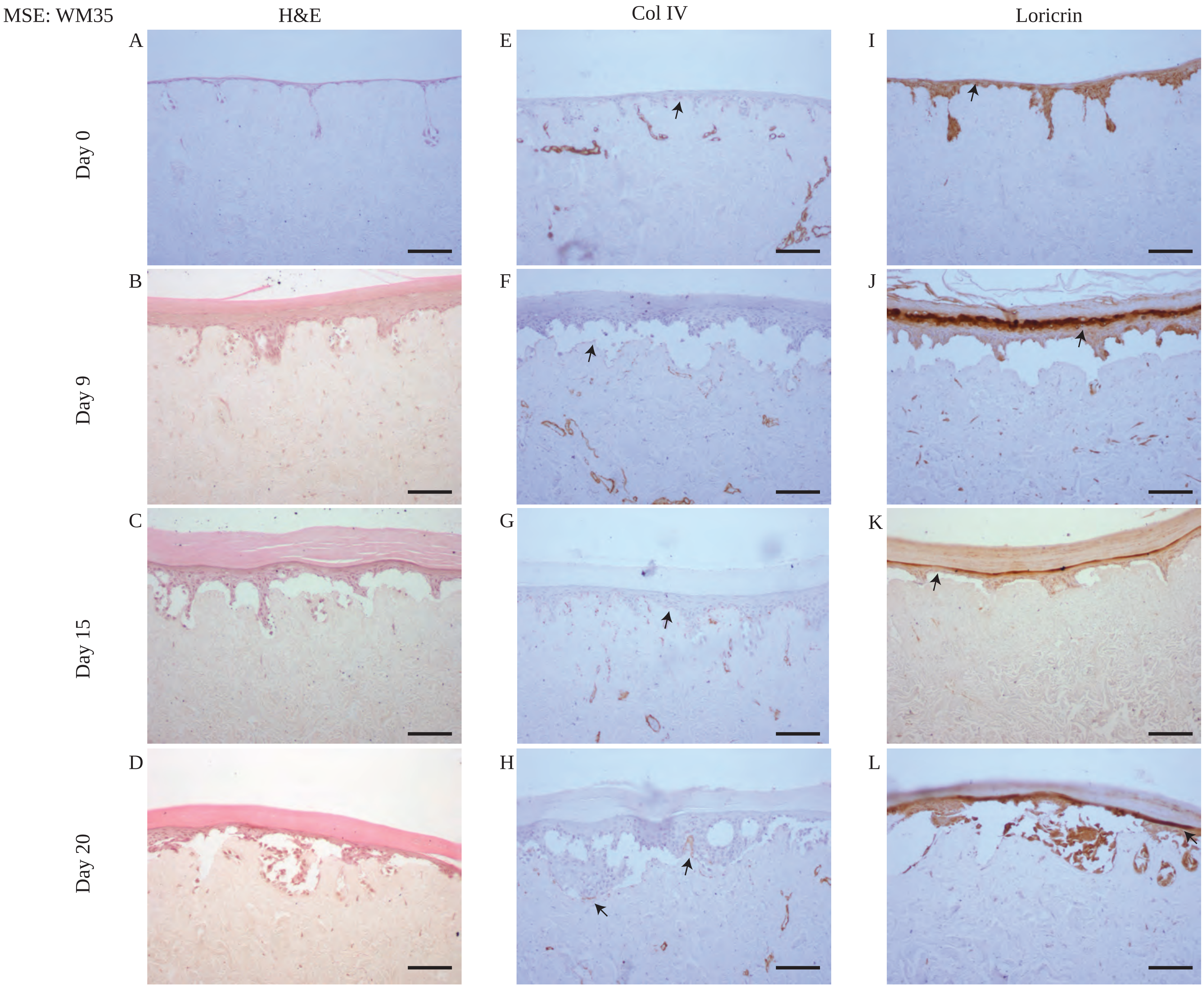

MSE: WM35
H&E
Col IV
Loricrin
Day 0
Day 9
Day 15
Day 20
A
B
C
D
E
F
G
H
I
J
K
L

Figure Caption S4: Histological analysis of MSE model with WM35 melanoma cells.

(A)-(D) H&E staining at day 0, 9, 15 and 20. (E)-(H) The basement membrane (brown) highlighted by collagen IV (Col IV) at day 0, 9, 15 and 20. (I)-(L) Terminally differentiating epithelial cells (brown) highlighted by loricrin at day 0, 9, 15 and 20. Black arrows indicate positive staining. Scale bar corresponds to 100 µm.

Histological analysis of MSE model with SK-MEL-28 melanoma cells

MSE models with SK-MEL-28 cells are first divided through the centre of the MTT positive region using a sterile blade and embedded in paraffin wax. The samples are then sectioned into 5 µm thick tissue sections. To show the physiological similarities of the MSE model to HSE and native skin we perform H&E staining, basement membrane staining using Col IV, and terminally differentiating epidermal cell staining using Loricrin, on these tissue sections, as shown in Fig. S5. Staining is performed at day 0, 9, 15 and 20 and the results are imaged using Olympus BX41 microscope fitted with an Olympus digital camera.

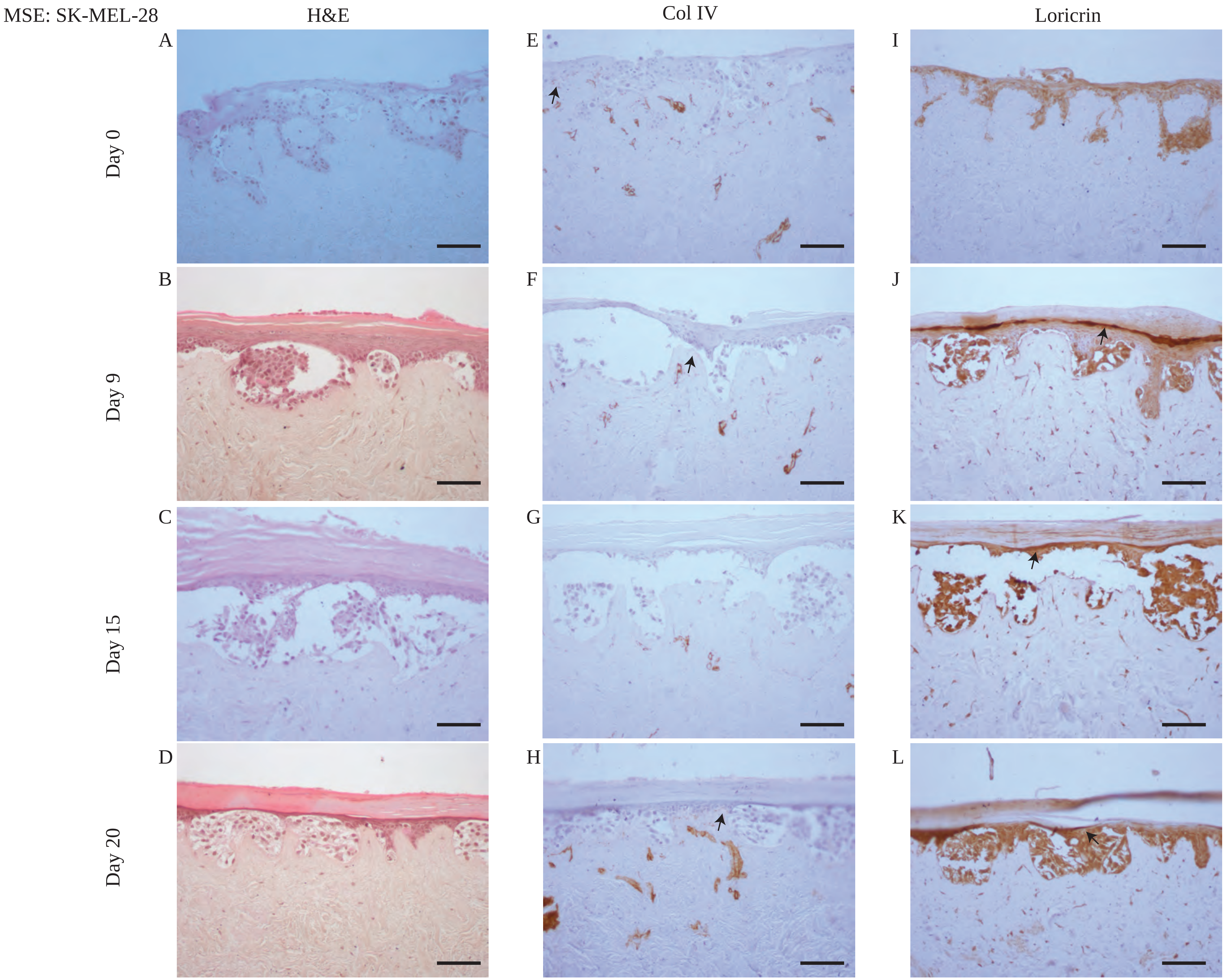

MSE: SK-MEL-28
H&E
Col IV
Loricrin
Day 0
Day 9
Day 15
Day 20
A
B
C
D
E
F
G
H
I
J
K
L

Figure Caption S5: Histological analysis of MSE model with SK-MEL-28 melanoma cells.

(A)-(D) H&E staining at day 0, 9, 15 and 20. (E)-(H) The basement membrane (brown) highlighted by collagen IV (Col IV) at day 0, 9, 15 and 20. (I)-(L) Terminally differentiating epithelial cells (brown) highlighted by loricrin at day 0, 9, 15 and 20. Black arrows indicate positive staining. Scale bar corresponds to 100 µm.

Estimating the size of melanoma cell lines

Images of melanoma cells are acquired using a brightfield microscope fitted with an Olympus digital camera. The size of each cell is measured using Leica LAS X software (http://www.leica-microsystems.com/applications/life-science/live-cell-imaging/). The average size of WM35 cells is approximately 9 μm and the average size of SK-MEL-28 cells is approximately 10 μm. These images show minimal differences between cell sizes for both melanoma cell lines. The average melanoma cell size is calculated using n=10 cells from each cell line.

A WM35 melanoma cells

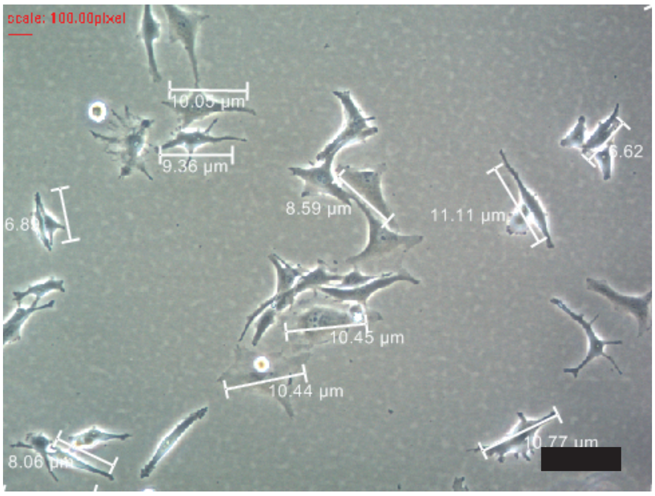

B SK-MEL-28 melanoma cells

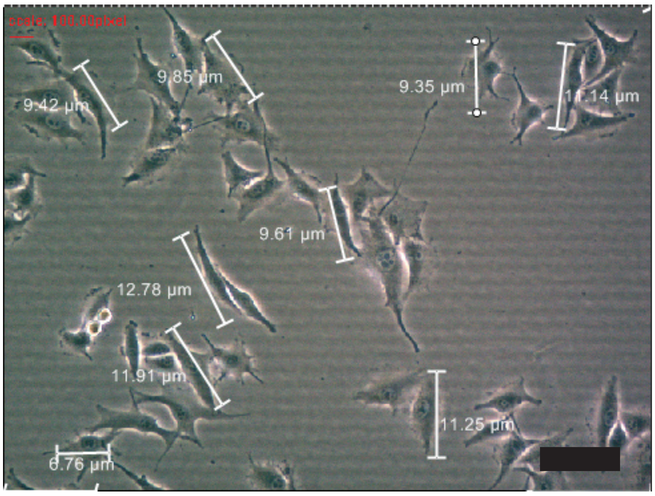

C

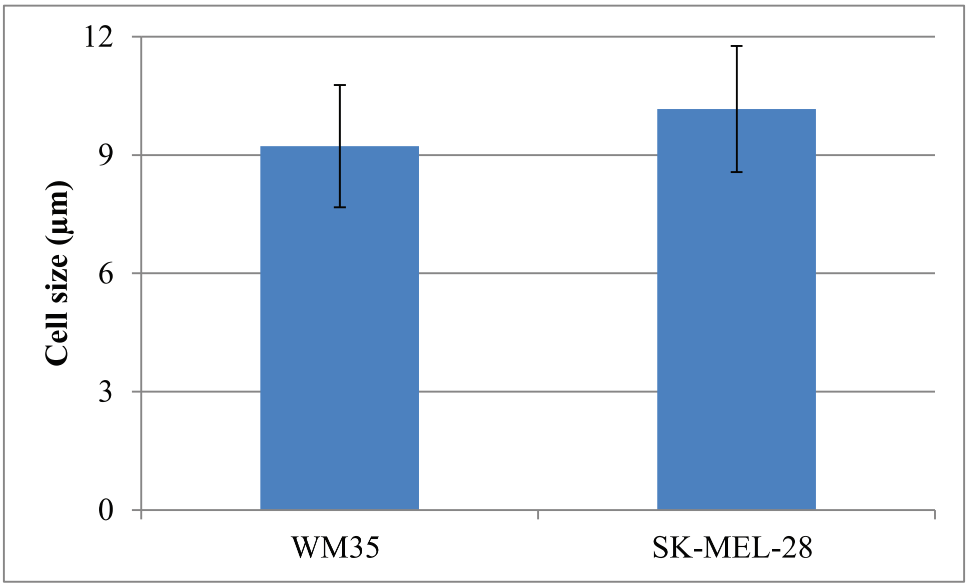

Figure Caption S6: Estimating the size of melanoma cell lines

(A) and (B) Images of melanoma cells, WM35 and SK-MEL28, showing cell size measurements. The scale bar corresponds to 10 µm. (C) Graphical representation of averaged cell sizes for WM35 and SK-MEL-28 melanoma cells. Averaged cell size is calculated using measurements from n=10 cells for each cell line. The error bars measure the variability as given by the standard deviation.
